# Serum Level of RIPK1/3 Correlated With the Prognosis in ICU Patients With Acute Ischemic Stroke

**DOI:** 10.1002/iid3.70085

**Published:** 2024-12-10

**Authors:** Jianhong Dong, Xinli Xiong

**Affiliations:** ^1^ Department of Intensive Care Unit, Beijing Boai Hospital China Rehabilitation Research Center Beijing China; ^2^ Department of Neurology, Shanghai East Hospital, School of Medicine Tongji University Shanghai China

**Keywords:** acute ischemic stroke, disease condition, prognosis, RIPK1, RIPK3

## Abstract

**Background:**

Acute ischemic stroke (AIS) is a common cerebrovascular disease with high mortality. AIS patients in the intensive care unit (ICU) often have severe conditions that require close monitoring and timely treatment. Receptor‐interacting protein kinase 1 (RIPK1) and RIPK3 play important roles in cell apoptosis and inflammation. However, the relevance of serum RIPK1/3 to AIS patients in the ICU has not been clarified.

**Objective:**

To explore the correlation of serum RIPK1 and RIPK3 with the prognosis of AIS patients in the ICU.

**Methods:**

One hundred and twenty AIS patients were selected as the research subjects for the retrospective analysis. The subjects were grouped based on the volume of cerebral infarction and the score of the National Institute of Health Stroke Scale (NIHSS) and mRS. The correlation was explored using Pearson analysis. The predictive value was valued using the ROC curve.

**Results:**

The content of serum RIPK1 and RIPK3 was gradually elevated with increased cerebral infarction volume and the severity of the disease (*p* < 0.05). Patients with poor prognosis had a higher content of serum RIPK1 and RIPK3 than those with good prognosis (*p* < 0.05). Serum RIPK1 and RIPK3 levels were positively correlated with infarct volume, NHISS, and mRS scores (*p* < 0.001). The area under the curve (AUC) of RIPK1 and RIPK3 for predicting the severity of AIS was 0.703, 0.883, and 0.912, respectively. The AUC for predicting poor prognosis of AIS was 0.797, 0.721, and 0.893, respectively. The cooperative detection of RIPK1 and RIPK3 had higher clinical value.

**Conclusion:**

AIS patients in the ICU had abnormally elevated content of serum RIPK1 and RIPK3, which was closely related to the volume of cerebral infarction, severity, and prognosis. Combined detection of RIPK1 and RIPK3 might help to early identify the severity and evaluate the prognosis, providing a reference basis for clinical doctors to develop treatment strategies.

## Graphical abstract

AIS patients in ICU had abnormally elevated content of serum RIPK1 and RIPK3, which was closely related to the volume of cerebral infarction, severity and the prognosis.

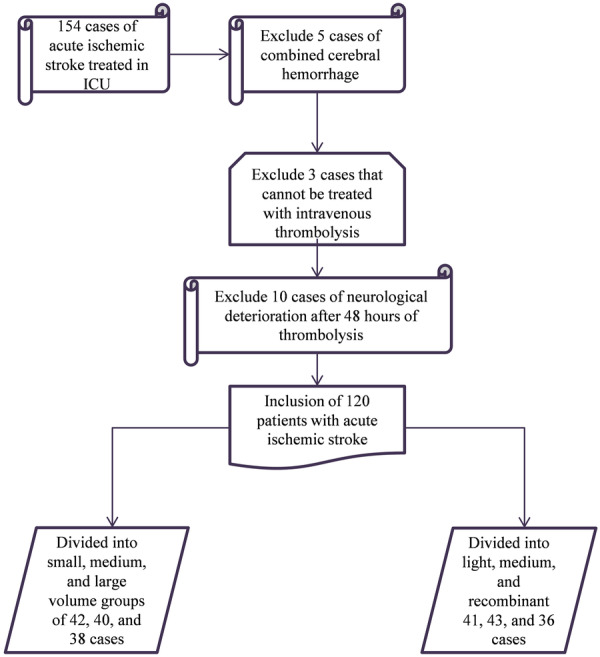

## 1 Introduction

Acute ischemic stroke (AIS) is a type of brain dysfunction disease induced by various reasons related to blood supply disorders in brain tissue and further ischemic and hypoxic necrosis. Stroke is one of the major diseases that lead to death and disability in humans, and is the third cause of death in China [1]. The morbidity of stroke in China shows no sign of waning, and the new cases account for up to one‐fourth of the global annual cases. The Global Burden of Disease (GBD) investigation showed that [2], China had 3.94 million newly emerged stroke patients in 2019, with a total of 28.76 million cases and 2.19 million stroke‐related deaths. Ischemic stroke is the most common type, accounting for over 80% of stroke patients [3]. In a large nationally representative sample survey of adults aged 40 and above, the projected prevalence, incidence, and mortality of stroke in China in 2020 are 2.6%, 505.2/100,000 people/year, and 343.4/100,000 people/year, respectively [4]. Moreover, the disability rates of Chinese AIS survivors at 3 months and 12 months are 14.8% and 14.0%, respectively, and the mortality rates of stroke at 3 months and 12 months are 4.2% and 8.5%, respectively. The recurrence rate of stroke at 3 months and 12 months are 3.6% and 5.6%, respectively [5]. The key to AIS treatment lies in the early opening of obstructed blood vessels and rescuing the ischemic penumbra, with standard intravenous thrombolysis as the most basic treatment method at present. Multiple guidelines recommend the application of intravenous Recombinant Tissue Plasminogen Activator (rt‐PA) within 4.5 h of ischemic stroke onset for patients with indications. However, for patients with large vessel occlusion, the success rate of vascular recanalization is less than 30%, and the 90‐day mortality rate and disability rate are 21% and 68%, respectively. The treatment effect is still far from satisfactory [6, 7]. Mechanical thrombectomy can help patients open blood vessels and provide oxygen support for ischemic brain tissue. However, studies have shown that cerebral hemorrhage is a common complication in mechanical thrombectomy, mainly caused by vascular intimal injury, ischemia‐reperfusion injury, thrombolytic drugs, antiplatelet, anticoagulant drugs, etc., which seriously affect postoperative recovery and increase the risk of patient death [8]. Early diagnosis and control of disease progression are necessary to improve the prognosis of AIS patients. It is difficult to accurately judge the severity of the patient's condition solely based on clinical symptoms. Therefore, searching for more accurate clinical biomarkers to evaluate the condition of AIS patients has become a research hotspot in recent years.

Research has shown that necroptosis is widely involved in the onset and progression of pathological mechanisms including stroke, myocardial reperfusion injury, tumors, immune inflammation, etc. Therefore, necroptosis has received much attention in recent years [9]. In the process of continuous in‐depth research, Receptor‐interacting protein kinase 1 (RIPK1) and RIPK3, members of the serine/threonine protein kinase family, were received with concern. Studies have shown that activation of RIPK1 in microglia can cause polarization of microglia into M1 phenotype, leading to increased death of oligodendrocytes and inflammation [10]. In the study of cerebral ischemia models [11], it was found that the degree of brain injury and neuronal death decreases when the phosphorylation of RIPK1/3 protein is inhibited. However, there are currently few research reports on the relationship between changes in serum RIPK1 and RIPK3 levels with AIS conditions.

In this study, the correlation between RIPK1 and RIPK3 with cerebral infarction volume, severity, and prognosis in AIS patients was explored, aiming to provide new targets for the treatment of AIS and bring better prognosis to patients.

## 2 Materials and Methods

### 2.1 General Material

**Figure 1:**
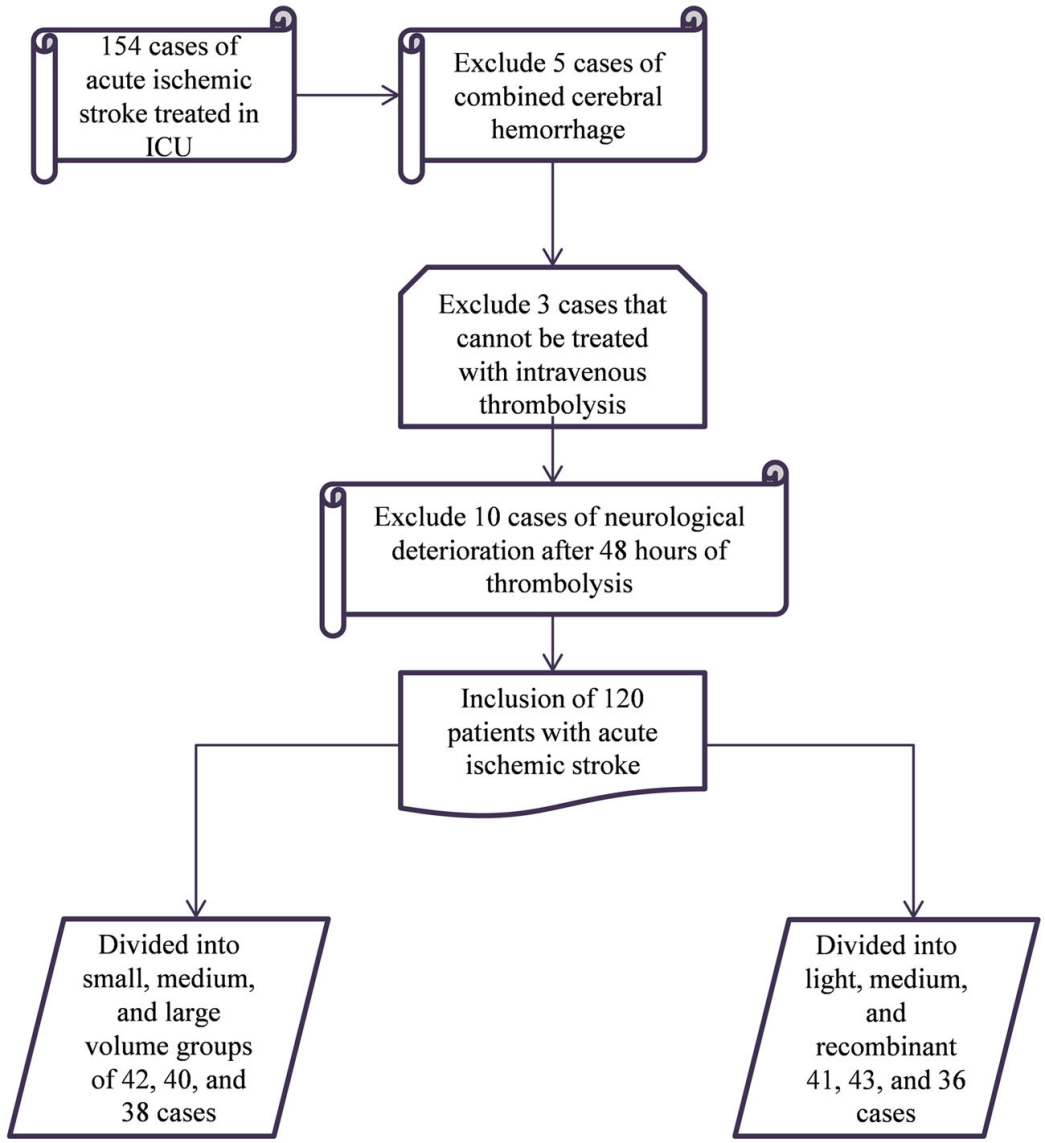
The inclusion process of patients.

A sum up of 120 AIS patients (63 males and 57 females aged 60–80 years) who received thrombolysis treatment in the ICU of our hospital from February 2021 to October 2022 were chosen as the study subjects. The clinical data of these subjects were retrospectively analyzed, and the inclusion process is shown in Figure 1. They had an average age of (68.72 ± 5.33) years, with 75 cases undergoing intravenous thrombolysis and 45 cases undergoing mechanical thrombectomy. Inclusion criteria: (1) All subjects conformed to the diagnostic criteria for AIS [12], and were further confirmed by Imaging examination; (2) Admission within 48 h of the first onset; (3) Those with a survival time of over 3 months; (4) A clear infarct lesion was confirmed by Imaging. Exclusion criteria: (1) Patients with combined Alzheimer's and Parkinson's disease; (2) Diagnosed cerebral hemorrhage through head CT; (3) Individuals with liver, kidney or heart dysfunction have a higher risk during the research process; (4) Individuals with incomplete medical record information; (5) Individuals with combined intracranial infection or malignant tumors in other areas; (6) There existed relative or absolute contraindications for intravenous thrombolysis treatment, and neurological deterioration occurred 48 h after thrombolysis; (7) Individuals with a history of traumatic brain injury or surgery; (8) Individuals with coagulation dysfunction and hematological disorders. In addition, 40 patients with carotid atherosclerotic plaque but no AIS who were admitted to our hospital during the same period were included as the control (24 males and 16 females aged 60–82 years, with an average age of 67.44 ± 4.39). The general data of the two groups were comparable (*p* > 0.05). This study has been ratified by the hospital Ethics Committee ([2021]−009) and has obtained written informed consent from patients.

### 2.2 Grouping

According to the volume of cerebral infarction [13], patients were grouped as a small volume group of 42 cases (infarct volume< 3 cm^3^), a medium volume group of 40 cases (infarct volume between 3 and 5 cm^3^), and a large volume group of 38 cases (infarct volume> 5 cm^3^). The calculation of cerebral infarction volume in ischemic stroke patients is based on the Pulicino formula=longest diameter of infarction displayed on head CT/MRI x the largest diameter x the number of layers x (interlayer distance + layer thickness) x 0.5. If there exist multiple infarctions, the total volume of scattered multiple infarctions is the summation of the total volume of each infarction.

The 120 patients were grouped abiding by the National Institute of Health Stroke Scale (NIHSS) score [14], namely as the severe group (36 cases with NIHSS > 16 points), the moderate group (43 cases with 5–15 points), and the mild group (41 cases with NIHSS < 5 points).

According to the mRS score followed up for 90 days [15], patients were further distinguished as the good prognosis group (72 cases with mRS score ≤ 2 points) and the poor prognosis group (48 cases with mRS score> 2 points).

### 2.3 Detection Methods

On the second day of hospitalization, 3 mL of fasting venous blood was drawn from the enrolled patients, while the control group received blood drawn during physical examination. The plasma was separated after centrifugation at 3000 r/min for 10 min and was stored at −80°C for subsequent testing. Plasma RIPK1, RIPK3, and tumor necrosis factor‐α (TNF‐α) were measured using a corresponding enzyme‐linked immunosorbent assay kit (Wuhan Yunclone Technology Co., Ltd., No.106528‐T08 and SEE639Hu).

### 2.4 Statistical Analysis

SPSS 20.0 software was employed for data analysis. Quantitative data were represented by ($\bar{x}$ ± *s*) and one‐way repeated measures ANOVA was used for intergroup comparisons. LSD *t*‐test was further used for pairwise intergroup comparisons. Enumeration data were indicated in (%), and compared using *χ* ^*2*^ inspection. The correlation between serum RIPK1 and RIPK3 content with infarct volume, NHISS, and mRS score was analyzed using Pearson analysis; The predictive value of serum RIPK1 and RIPK3 for the severity and prognosis of AIS was assessed using ROC curve analysis. The statistical results were statistically significant in the cases of *p* < 0.05.

## 3 Results

### 3.1 Serum RIPK1, RIPK3, and TNF‐α Levels With Cerebral Infarction Volumes

Serum RIPK1, RIPK3, and TNF‐α levels between the control group and different cerebral infarction volume groups were compared using ANOVA. RIPK1, RIPK3 and TNF‐α concentrations among the four groups had statistically significant differences (F = 263.13, *p* < 0.001; F = 5204.10, *p* < 0.001; F = 216.70, *p* < 0.001). The pairwise comparison showed that those with different cerebral infarction volumes all had a much higher content of serum RIPK1, RIPK3, and TNF‐α than the control (*p* < 0.05). Besides, the medium and large volume groups had markedly higher content of RIPK1, RIPK3, and TNF‐α than the small volume group (*p* < 0.05). The large volume group had a higher content of RIPK1, RIPK3, and TNF‐α than the medium volume group (*p* < 0.05, Table 1).

**Table 1:** Comparison of serum RIPK1, RIPK3, and TNF‐α levels in patients with different volumes of cerebral infarction.

Groups	Cases	TNF‐α(μg/L)	RIPK1(μg/mL)	RIPK3(μg/mL)
The control group	40	2.87 ± 1.10	1.93 ± 0.18	0.60 ± 0.13
The small volume group	42	5.21 ± 2.17	3.55 ± 0.20*	9.08 ± 1.52*
The medium volume group	40	10.70 ± 1.56	5.48 ± 2.23* ^,^ **	25.66 ± 1.87* ^,^ **
The large volume group	38	16.18 ± 4.23	8.79 ± 0.34* ^,^ ** ^,^ ***	44.90 ± 2.42* ^,^ ** ^,^ ***
*F*		216.70	263.13	5204.10
*P*		< 0.001	< 0.001	< 0.001

* *p* < 0.05 compared with the control group

** *p* < 0.05 compared with the small volume group

*** *p* < 0.05 compared with the medium volume group.

### 3.2 Serum RIPK1, RIPK3, and TNF‐α Levels With Disease Severity

The difference in serum RIPK1, RIPK3, and TNF‐α content between the control group and different disease severity groups was analyzed using ANOVA. RIPK1, RIPK3 and TNF‐α concentrations among the four groups had statistically significant differences (F = 2496.30, *p* < 0.001; F = 3317.25, *p* < 0.001; F = 63.69, *p* < 0.001). The pairwise comparison showed that patients with different disease severity had a much higher content of serum RIPK1, RIPK3, and TNF‐α than the control (*p* < 0.05). The moderate and severe groups had a markedly higher content of RIPK1, RIPK3, and TNF‐α than the mild ones (*p* < 0.05). As expected, the severe group had a higher content of RIPK1, RIPK3, and TNF‐α than the moderate group (*p* < 0.05, Table 2).

**Table 2:** Comparison of serum RIPK1, RIPK3, and TNF‐α levels in patients with different disease severity.

Groups	Cases	TNF‐α(μg/L)	RIPK1 (μg/mL)	RIPK3 (μg/mL)
The control group	40	2.87 ± 1.10	1.93 ± 0.18	0.60 ± 0.13
The mild group	41	7.72 ± 3.85	3.80 ± 0.20*	10.39 ± 0.56*
The moderate group	43	14.70 ± 5.12	5.68 ± 0.36* ^,^ **	21.67 ± 1.88* ^,^ **
The severe group	36	17.79 ± 8.56	7.52 ± 0.40* ^,^ ** ^,^ ***	42.10 ± 3.39* ^,^ ** ^,^ ***
*F*		63.69	2496.30	3317.25
*P*		< 0.001	< 0.001	< 0.001

* *p* < 0.05 compared with the control group

** *p* < 0.05 compared with the mild group

*** *p* < 0.05 compared with the moderate group.

### 3.3 Serum RIPK1, RIPK3, and TNF‐α Levels With Prognoses

Patients with poor prognosis had significantly higher serum RIPK1, RIPK3, and TNF‐α content than those with good prognosis (*p* < 0.05, Table 3).

**Table 3:** Comparison of serum RIPK1, RIPK3, and TNF‐α levels in patients with different prognoses.

Groups	Cases	TNF‐α(μg/L)	RIPK1 (μg/mL)	RIPK3 (μg/mL)
Good prognosis group	72	10.09 ± 4.94	4.25 ± 0.34	9.36 ± 1.26
Poor prognosis group	48	20.10 ± 8.48	7.89 ± 0.52	39.41 ± 3.87
*t*		8.161	46.398	61.301
*P*		< 0.001	< 0.001	< 0.001

### 3.4 The Correlation Between Serum RIPK1, RIPK3, and TNF‐α With Infarct Volume, NHISS, and mRS Scores

**Figure 2:**
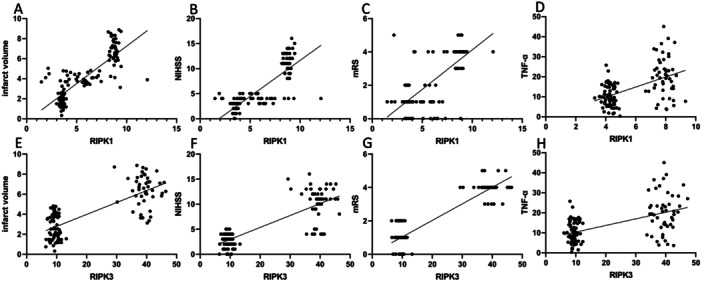
The correlation between serum RIPK1 and RIPK3 levels and infarct volume, NHISS, mRS scores and TNF‐α level. (A) The correlation between serum level of RIPK1 and infarct volume; (B) The correlation between serum level of RIPK1 and NIHSS score; (C) The correlation between serum level of RIPK1 and mRS score; (D) The correlation between serum level of RIPK1 and TNF‐α level; (E) The correlation between serum level of RIPK3 and infarct volume; (F) The correlation between serum level of RIPK3 and NIHSS score; (G) The correlation between serum level of RIPK3 and mRS score; (H) The correlation between serum level of RIPK3 and TNF‐α level.

Serum RIPK1, RIPK3, and TNF‐α contents were positively correlated with infarct volume, NHISS, and mRS scores, respectively (*p* < 0.001, Table 4 and Figure 2).

**Table 4:** The correlation between serum RIPK1, RIPK3, and TNF‐α levels and infarct volume, NHISS, and mRS scores.

Indicators	RIPK1		RIPK3	
	*r*	*P*	*r*	*P*
Infarct volume	0.815	< 0.001	0.795	< 0.001
NHISS score	0.820	< 0.001	0.689	< 0.001
mRS score	0.729	< 0.001	0.774	< 0.001
TNF‐α	0.519	< 0.001	0.554	< 0.001

### 3.5 The Predictive Value of Serum RIPK1 and RIPK3 for the Severity of AIS

**Figure 3:**
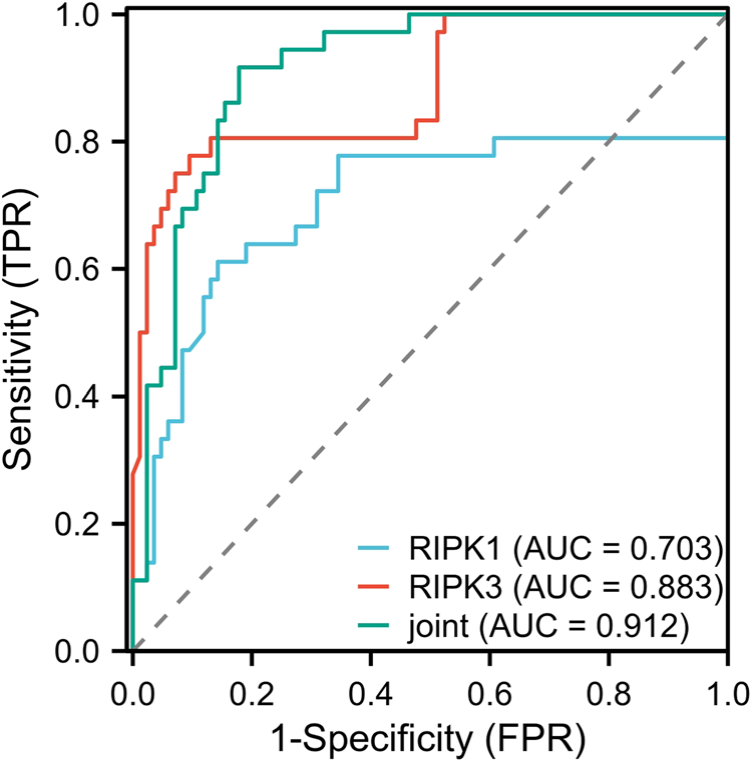
The predictive value of serum RIPK1 and RIPK3 levels for the severity of AIS.

ROC curve analysis took the disease condition as the dependent variable (0=mild to moderate, 1=severe), and serum RIPK1 and RIPK3 as independent variables (continuous variables). The AUC of individual or joint RIPK1 and RIPK3 for predicting the severity of AIS was 0.703, 0.883, and 0.912, respectively (Table 5 and Figure 3).

**Table 5:** The predictive value of serum RIPK1 and RIPK3 levels for the severity of AIS.

Indicators	AUC	95% CI	Sensitivity (%)	Specificity (%)	Cut‐off value	Youden's index
RIPK1	0.703	0.577–0.830	61.11	85.71	5.9 μg/mL	0.468
RIPK3	0.883	0.813–0.954	77.78	90.48	22.92 μg/mL	0.683
Combined detection	0.912	0.861–0.963	91.67	82.14	/	0.738

### 3.6 The Predictive Value of Serum RIPK1 and RIPK3 for Prognosis in AIS

**Figure 4:**
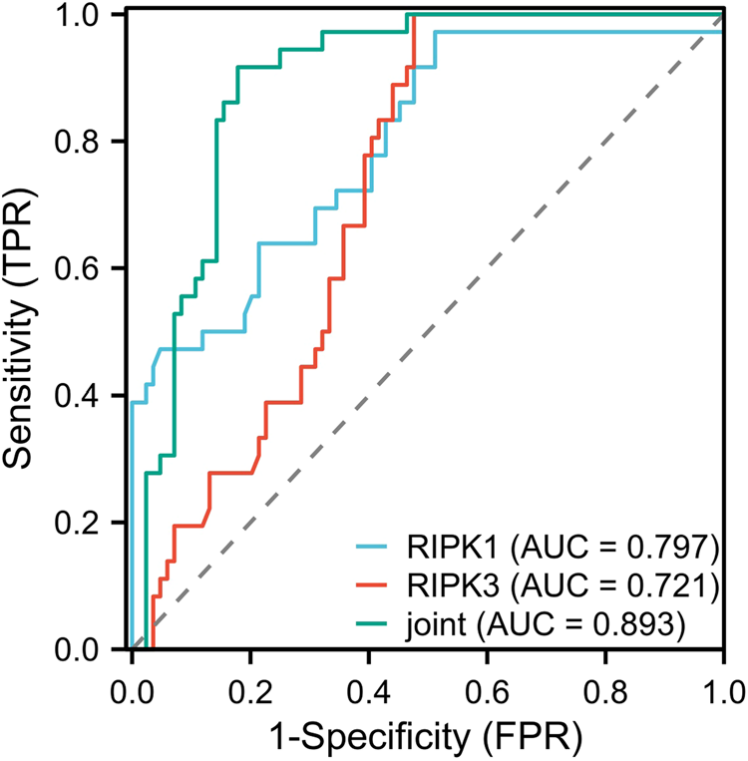
The predictive value of serum RIPK1 and RIPK3 levels for poor prognosis in AIS.

According to the mRS score of 90 days of follow‐up, patients with ≤ 2 and > 2 scores were taken as the good and the poor prognosis group, respectively. The predictive value of serum RIPK1 and RIPK3 for prognosis was analyzed using ROC analysis. The AUC of individual or joint RIPK1 and RIPK3 levels for predicting poor prognosis in AIS were 0.797, 0.721, and 0.893, respectively (Table 6 and Figure 4).

**Table 6:** The predictive value of serum RIPK1 and RIPK3 levels for poor prognosis in AIS.

Indicators	AUC	95% CI	Sensitivity (%)	Specificity (%)	Cut‐off value	Youden's index
RIPK1	0.797	0.709‐0.885	97.22	48.88	6.27	0.460
RIPK3	0.721	0.632‐0.809	88.23	80.48	18.92	0.687
Combined detection	0.893	0.836‐0.950	91.67	82.14	/	0.738

## 4 Discussion

AIS has a high incidence rate and recurrence rate, which is an important reason related to clinical death and disability, and brings a heavy economic burden to both the families and society. The onset of AIS is time‐sensitive and widespread. The imaging examination results and information provided during emergency visits are key influencing factors for clinical physicians to make optimal treatment decisions, such as the area of the infarct core and ischemic penumbra, the presence and location of intravascular thrombosis, the risk and probability of bleeding transformation, prediction and improvement of prognosis, etc [16]. In this study, we compared potential confounding variables such as age and gender between AIS patients and a control group, and AIS patients were matched into a control group with similar characteristics such as age and gender, so as to control the impact of these confounding variables on research results.

Cerebral ischemia‐reperfusion (I/R) injury refers to the restoration of blood supply after cerebral ischemia, which not only fails to restore brain function but also leads to more serious brain dysfunction [17]. I/R injury is related to the accumulation of free radicals, overloaded intracellular calcium, excitatory amino acid toxicity, excessive leukocyte aggregation, and deficiency of high‐energy phosphate compounds [18]. Necroptosis is a programmed cell death process that does not rely on Caspase activation, but the production of necrotic bodies. Necrotic apoptosis has a typical necrotic morphology, including cell membrane damage, swelling of cells and organelles, and even disintegration. No significant morphological change occurs in the chromatin within the nucleus. Secondly, the inflammatory response caused by necrotic apoptosis is characterized by extensive infiltration and activation of inflammatory cells [19]. Serum RIPK1 and RIPK3 are key protein molecules in programmed necrosis. When the death ligand binds to the death receptor, it further activates the RIPK1‐RIPK3‐MLKL pathway, leading to programmed necrosis [20]. More and more studies have shown that [21, 22], programmed necrosis is involved in the onset and progression of diversified cardiovascular diseases, such as I/R injury, atherosclerosis, heart failure, etc. Animal experiments and clinical studies have shown [23, 24] that RIPK1 and RIPK3 are core factors involved in the pathological processes of cell necrosis and apoptosis. However, few clinical studies are reporting the involvement of RPK1 and RPK3 in neuronal pathological processes after cerebral infarction. A study found [25] that in a mouse model of ischemic stroke, RPK3 deficiency could reduce lower volume, neurological deficits, and neuronal ultrastructural damage. Scholars have also found that the production of insoluble RIPK1, RPK3, and MLKL in the infarcted area of mice is elevated. In contrast with wild‐type mice, RIPK1 kinase‐dead mice, RIPK3 deficient mice, and MLKL deficient mice showed reduced levels of cell necrosis and neuroinflammation after cerebral ischemia, which is highly consistent with the apoptotic pathway of necrotic cells. These above results indicated that RIPK1 and RIPK3 mediated apoptosis of necrotic cells was involved in brain injury after AIS [26]. Therefore, RIPK1 and RIPK3 are closely related to the onset and progression of atherosclerosis and inflammation‐mediated stroke. After the stroke, brain tissue will undergo a series of complex pathophysiological changes, including ischemia, reperfusion injury, inflammation, etc. These changes are particularly significant in the early stage after stroke and may reach a peak [27]. Therefore, choosing a representative point in time to detect can help capture the impact of these changes on RIPK1 and RIPK3 levels. In this study, serological indexes of patients were detected on the second day after hospitalization. Our present study showed that AIS patients with different infarct sizes and severity groups had higher serum RIPK1 and RIPK3 levels than the control, indicating the existence of apoptotic pathway‐mediated cell necrosis pathway after AIS. In addition, the results of this study also revealed that the content of serum RIPK1 and RIPK3 was positively correlated with infarct volume, NHISS, and mRS scores, respectively. Early studies have confirmed [28] that the wider the infarct area of acute stroke was, the more severe the symptoms of neurological deficits were, and the more obvious the inflammatory response was. Therefore, serum RIPK1 and RIPK3 levels can reflect the severity of inflammation expression and the severity of the condition after stroke. In addition, studies have also found [29] that inhibiting RIPK1 and RIPK3 levels in ischemic stroke patients can reduce infarct volume and improve the degree of neurological deficits, indirectly confirming that poor prognosis in stroke patients may be related to abnormal expression of RIPK1 and RIPK3 levels.

After 90 days of follow‐up, it was found that patients with poor prognosis had much higher serum levels of RIPK1 and RIPK3 than those with good prognosis, suggesting that the increase of serum RIPK1 and RIPK3 levels may increase the risk of poor prognosis in AIS patients. The ROC curve analysis showed that the AUCs of serum RIPK1 and RIPK3 alone and in combination for predicting the severity of AIS were 0.703, 0.883, and 0.912, respectively. The AUCs for predicting poor prognosis in patients were 0.797, 0.721, and 0.893, respectively. Combined detection had the highest clinical value. From this, it could be seen that serum RIPK1 and RIPK3 had high predictive value in the assessment of the condition and prognosis of stroke. It has been reported that the predictive AUC of AIS's poor prognosis based on the nomogram model built on the whole‐body immunoinflammatory index is 0.657 [30]. In addition, the AUC values for predicting the prognosis of AIS patients at 3 months, 6 months, 12 months, and 18 months are 0.706, 0.653, 0.616, and 0.610 [31]. The AUC value of this study was slightly higher than that of other studies, which further confirmed the results of this study provided new insights for the diagnosis, evaluation, and treatment of AIS. The detection of serum RIPK13 levels may help to early identify the severity of the disease and provide an important reference for clinical doctors. In addition, treatment strategies targeting RIPK13 may bring breakthroughs in the treatment of AIS.

In general, AIS patients in the ICU had abnormally elevated content of serum RIPK1 and RIPK3, which were closely related to the volume of cerebral infarction, severity, and prognosis. Combined detection of RIPK1 and RIPK3 might help to provide a reference basis for clinical doctors to develop treatment strategies. However, this study also had some limitations.

## 5 Novelty

Although the role of RIPK1 and RIPK3 in the regulation of apoptotic signaling has been extensively studied, this study may delve deeper into the specific correlation between RIPK1 and RIPK3 and specific levels in the serum of stroke patients. For example, we measured serum levels of RIPK1 and RIPK3 in AIS patients to reveal specific associations between these kinase levels and disease severity and prognosis, which may not have been fully explored in previous studies. Although the mechanisms of necrotic apoptosis in ischemic stroke have been explored, this study further deepened the understanding of stroke pathogenesis by focusing on the specific roles of RIPK1 and RIPK3, such as revealing how RIPK1 and RIPK3 affect the inflammatory response after stroke, the process of cell death, and the resilience of neurons. Thus, new therapeutic targets or intervention strategies are provided. This study may not only reveal the association of RIPK1 and RIPK3 with stroke severity and prognosis but also explore how these findings can be translated into clinical applications. In addition, we propose new diagnostic methods, prognostic assessment tools, or treatment strategies based on RIPK1 and RIPK3 levels that have the potential to bring practical medical benefits to stroke patients.

## 6 Limitation

First, because the sample size is limited and only from a single center, there is a risk of limited universality and inclusion bias. This weakens the reliability and popularization value of the research results to some extent.

Secondly, this study did not dynamically observe the levels of RPK1 and RIPK3 in the patient's serum due to the time of the study. Subsequent multi‐center studies with large sample sizes are needed to verify our conclusion. We will also continue tracking the serum levels of RPK1 and RIPK3 in patients at 3, 6, and 12 months after discharge to improve the credibility of our conclusion. This study did not supplement the existing diagnostic gold standard in the ROC curve, which is a major limitation. At the same time, we may also consider combining other serological indicators or imaging findings to more comprehensively assess the severity and prognosis of cerebral infarction. In addition, since the patients included in this study are only AIS patients in our hospital, whether it will increase the risk of poor prognosis for all AIS, we still need to conduct a larger, multi‐center study to confirm.

In future studies, we would increase the sample size, adopt a multi‐center design, and strictly control the inclusion criteria to improve the universality and accuracy of the study. In addition, the retrospective design of this study inherently limits the ability of the study to establish causality and is susceptible to bias such as selection bias and recall bias. Therefore, when interpreting and applying the findings, we need to consider these limitations and make a comprehensive assessment in the context of other evidence. In the future, we can use prospective design or more rigorous retrospective analysis methods to further improve the accuracy and reliability of the study. In addition, this study shows that RIPK1 and RIPK3 have potential clinical applications as biomarkers, but the evidence provided is insufficient to support changing clinical practice, so further large‐scale, multicenter studies are needed to verify these findings.

## Author Contributions

**Jianhong Dong:** conceptualization, data curation, formal analysis, investigation, methodology, resources, writing–original draft. **Xinli Xiong:** methodology, project administration, resources, software, supervision, writing–review and editing.

## Ethics Statement

This study was ratified by The Ethics Committee of Beijing Boai Hospital ([2021]−009). Informed consent was obtained from participants for the participation in the study and all methods were carried out in accordance with relevant guidelines and regulations.

## Consent

The authors have nothing to report.

## Conflicts of Interest

The authors declare no conflicts of interest.

## Data Availability

The datasets used and/or analyzed during the current study are available from the corresponding author upon reasonable request.
